# Study of Missing Clinical Details in Computed Tomography Radiology Request Forms: A Descriptive Cross-sectional Study

**DOI:** 10.31729/jnma.4674

**Published:** 2020-02-29

**Authors:** Muna Badu

**Affiliations:** 1 Department of Radiology, Kathmandu Medical College and Teaching Hospital, Sinamangal, Kathmandu

**Keywords:** *data*, *forms*, *missing*, *Tomography*, *radiology.*

## Abstract

**Introduction:**

Detailed clinical history through a properly filled requisition form can help a radiologist in making a diagnosis. The objective of this study was to observe the missing clinical details of Computed Tomography requisition forms at radiology department in tertiary care hospital.

**Methods:**

This descriptive cross-sectional study was done in 196 Computed Tomography requisition forms in the department of radiology from September 2019 to October 2019. Ethical clearance from the Institutional Review Committee - Reference No. 120720194 was obtained. An informed consent from the participants was taken prior to the procedure. Convenient sampling was done. The data obtained were computed and analyzed using Statistical Package for Social Sciences to tabulate the results. The results were displayed in frequency and proportion of binary data.

**Results:**

All the request forms had name filled, however date was filled in 183 (93.4%), age was filled in 195 (99.5%), sex was filled in 193 (98.5%) and address was only in 30 (15.3%) of the forms. Clinical history and provisional diagnosis were written in 179 (91.3%) forms. Signature was found in more than half of forms 135 (68.9%) whereas the department referring the patient was filled in 92 (46.9%) of forms and the name of doctor referring the patient was not filled mostly. The handwriting was clear in 191 (97.4%) of cases and standard words were used. Use of non-standard abbreviation was found in only 2 (1%) forms.

**Conclusions:**

Clinical details were filled in most of the requisition forms however other parameters were still incompletely and inadequately filled.

## INTRODUCTION

Radiologists report informally that missing clinical information is a daily occurrence and is a constant source of irritation and delay in giving computed tomography (CT) reports.^1^ A radiologist helps in making a diagnosis. This can only be achieved if the clinicians give a detailed clinical history through a properly filled request form. These are documented requests filled by clinicians with demographic details, clinical history, provisional diagnosis and referring physician’s signature.^2^

Complete filling of the forms is important for radiologists to give concise diagnosis and avoid unhelpful examinations and radiation exposure.^3^ Inadequate information can reduce the value of the report and leading to a mistake in patient identification and delay in returning reports to correct destination.^4^

The objective of this study was to find out the prevalence of adequate filling of request forms of CT scans in radiology department of Kathmandu Medical College and Teaching Hospital.

## METHODS

This descriptive cross-sectional study was carried out on CT requisition forms from September 2019 to October 2019 in the department of radiology of Kathmandu Medical College and Teaching Hospital. Ethical clearance from the Institutional Review Committee - Reference No. 120720194 was obtained. Informed consent of the participants was taken prior to the procedure. All the details in the requisition forms were recorded. All the CT scan forms were included for study. Patients who did not give consent were not included in this study. Convenient sampling was done and the sample size was calculated with prevalence 50%.

The sample size (n) was calculated as follows:

n = Z^2^ × p × q/e^2^

   = (1.96)^2^x0.5x0.5/(0.07)^2^

   = 196

Where,

n= Sample size
Z= 1.96 for 95% confidence interval
p = prevalence (50%)
q = 1-p
e= margin of error, 7 %

Hence, the total sample size taken was 196.

The data obtained were computed and analyzed using SPSS to tabulate the results. The results were displayed in frequency and proportion of binary data.

## RESULTS

A total of 196 CT request forms were studied for their completion. All the request forms had name filled, however date was filled in 183 (93.4%), age was filled in 195 (99.5%), sex was filled in 193 (98.5%) and address was only in 30 (15.3%) of the forms (Table 1).

**Table 1:** Demographic profile.

S.N.	Variables	n (%)
1.	Date	183 (93.4)
2.	Patient’s Name	196 (100)
3.	Age	195 (99.5)
4.	Sex	193 (98.5)
5.	Address	30 (15.3)

**Figure 1:**
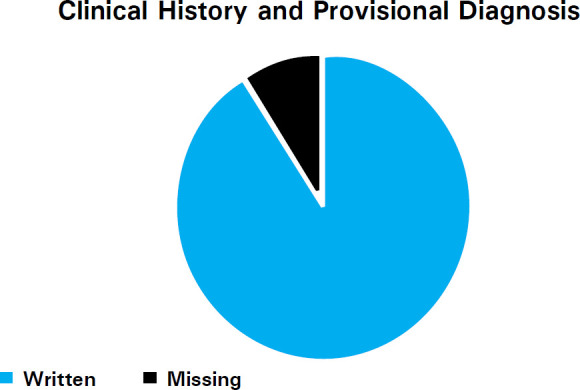
Complete Clinical History and Provisional Diagnosis in requisition forms.

Clinical history and provisional diagnosis were written in 179 (91.3%) forms and it was missing in 17 (8.67%) requisition forms (Figure 1).

Signature was found in more than half of forms 135 (68.9%) whereas the department referring the patient was filled in 92 (46.9%) of forms and the name of doctor referring the patient was not filled mostly (Table 2).

**Table 2:** Referral details.

S.N.	Referral details	n (%)
1.	Referred by	60 (30.6)
2.	Department	92 (46.9)
3.	Signature	135 (68.9)

The handwriting was clear in 191 (97.4%) of cases and standard words were used. Use of non-standard abbreviation was found in only 2 (1%) forms (Table 3).

**Table 3:** The number and percentage of request forms with non-illegible handwriting and non-standard abbreviations.

S.N.	Variables	n (%)
1.	Legibility of handwriting	191 (97.4)
2.	Use of non-standard abbreviation	2 (1)

## DISCUSSION

The radiology requisition forms are usually the only means of communication between clinician and radiologists.^5^ The opportunity to discuss cases between clinician and radiologist is not always possible. Whenever necessary, additional information can be acquired by radiologist by contacting patient or referring physician.^6^

Incomplete filling of radiology request forms is a worldwide problem.^7^ The Royal College of Radiologists clearly suggested that all radiology request forms should be adequately and legibly completed to avoid any misunderstanding of the request.^8^ The level of information in radiology request forms reflect the quality of radiology reports.^9^

Inadequate information in demographic details may affect in identification of the patient. Patient name was filled in all cases (100%) which is similar to studies done by Anjum et al, Afolabi et al and Irurhe et al.^3,7,10^ Date was filled in 93.4 % of cases which is close to study done by Afolabi et al and Irurhe et al.^7,10^ Age of the patient was filled in 99.5% of cases which is similar to observation made by Akinola et al. and Irurhe et al.^6,10^ However Jumah et al. found that the age of the patient was not filled mostly.^11^ Sex was mentioned in 98.5% of cases which is close to studies done by Irurhe et al. and higher than in study by Anjum et al.^3,10^ Address was filled only in 15.3% of forms in my study which is close to study done by Irurhe et al, Afolabi et al. and Anjum et al.^3,7,10^

Clinical details are one of the most important part of the radiology request forms. Inadequate and missing clinical details may lead to inaccurate report while accurate clinical information help radiologist in making a report which may be more helpful to referring doctor and patient management.^6^ In my study clinical details were filled in 91.3% of cases which is similar to studies done by Akinola et al and Irurhe et al.^6,10^ In studies done by Afolabi et al, Abbas et al and Jumah et al clinical details were inadequate.^7,12,13^

The name of the referral doctor, referral department and signature of the doctor were found in 30.6%, 46.9% and 68.9% of request forms respectively. This finding is lower compared to observation made by Abbas et al. and Irurhe et al.^10,12^

Illegible handwriting and use of non-standard abbreviations were found in 2.6% and 1% of cases. This finding is least common compared to previous studies by Akintomide et al, Rao and Jumah et al.^11,13,14^

The observation of missing clinical details in CT requisitions had different results in different studies. Some found it be less while other found it be high. Most of the requisition forms in my study had clinical details written on it with legible handwriting and very less use of non-standard abbreviations.

The sample size was small in this study which might make the results less generalizable. Information like last menstrual period, renal function tests, allergic history and current usage of drugs like metformin are very important factors for consideration for CT scan which were missing in the requisition form of our hospital.

## CONCLUSIONS

Clinical details were filled in most of the requisition forms however other parameters were still incompletely and inadequately filled.
